# Effects of the anti-oxidant PDTC in combination with a single bout of treadmill running on murine skeletal muscle

**DOI:** 10.1080/13510002.2020.1807088

**Published:** 2020-08-18

**Authors:** Angelika Schmitt, Anne-Lena Brändle, Pascal Herzog, Franziska Röchner, Annunziata Fragasso, Barbara Munz

**Affiliations:** a Department of Sports Medicine, University Hospital Tübingen, Medical Clinic, Tübingen, Germany; b Interfaculty Research Institute for Sport and Physical Activity, Eberhard Karls University of Tübingen, Tübingen, Germany

**Keywords:** Mice, skeletal muscle, running, PDTC, ROS, exercise, training adaptation, anti–oxidants

## Abstract

**Objectives:** Skeletal muscle adaptation to physical activity is dependent on various factors. Important signaling mediators are reactive oxygen species (ROS). However, recent research suggests that ROS have both beneficial and deleterious effects on exercise adaptation, dependent on training intensity and training status, so that the question of whether anti-oxidants should be taken in connection with exercise cannot easily be answered. Thus, it is important to gain more insight into the complex roles of ROS in regulating training adaptation.

**Methods:** The effects of ROS inhibition on skeletal muscle training adaptation were analyzed by applying the anti-oxidant PDTC, which is also an inhibitor of the ROS-activated transcription factor nuclear factor kappa B (NFκB), to juvenile mice in connection with a single bout of treadmill running.

**Results:** We found that PDTC inhibits exercise-mediated induction of specific stress- and inflammation-associated genes. Other genes, specifically those encoding metabolic and mitochondrial factors, were affected to a lesser extent and there appeared to be little effect on the microRNA (miR) profile.

**Discussion:** Our data suggest that anti-oxidants regulate distinct sets of adaptation-relevant genes, which might have important implications for the design of exercise-based preventive and therapeutic approaches.

## Introduction

Physical activity induces characteristic adaptation reactions in skeletal muscle tissue. The molecular signals that initiate these reactions are diverse, ranging from mechanical signals such as muscle fiber and sarcomere stretching, Ca^2+^ oscillations with muscle contraction, energy depletion, such as a decreasing ATP/ADP ratio, or systemic factors, such as growth factors and hormones (for review, see [1]).

It has been known for a long time that exercise induces the production of reactive oxygen species (ROS), both from non-muscle sources, such as inflammatory cells, and the muscle cells themselves, for example as a result of mitochondrial ‘leakage’. While ROS might have deleterious effects, particularly by inducing muscle atrophy, more and more results suggest that they might also be beneficial and – to a certain extent – necessary to allow exercise adaptation. This effect appears to be the result of ROS acting as signaling mediators, for example by activating the MAPK signaling pathway or transcription factors nuclear factor erythroid 2-related factor 2 (Nrf2), or nuclear factor kappa B (NFκB). Whereas in trained individuals, the beneficial effects of ROS appear to largely outweigh the unwanted ones, most likely as a result of a training-induced more active anti-oxidant defense system, the situation in untrained individuals is more complex. In particular, it is still not entirely clear which adaptation-relevant genes are regulated by ROS in response to an unaccustomed bout of exercise. A detailed answer to this question, however, is of high practical relevance, since it would allow the development of recommendations with regard to consumption of anti-oxidants in the context of particular training regimens. In addition, since different anti-oxidants can target particular types of ROS in a specific manner, it is important to understand their unique effects in the context of defined exercise settings (for review, see [2–4]).

To get more insight into these complex interrelationships, for the study described here, mice were treated with PDTC (pyrrolidine dithiocarbamate). Dithiocarbamates are anti-oxidants that also display a strong NFκB-inhibiting effect (for review, see [5]), mainly due to a blockade of IκBα (inhibitor kappa B alpha) degradation [6–8]. After PDTC treatment, mice were then subjected to a single bout of endurance exercise (one hour of treadmill running). Subsequently, activity of signal transduction pathways and gene expression patterns in skeletal muscle were studied, with a specific focus on markers of inflammation, cellular stress, and mitochondria and metabolism.

These markers were chosen mainly based on previous work, focusing on differential gene expression in response to one single, acute bout of endurance exercise ([9]; for review, see [2,10]). Specifically, with regard to cell stress and inflammation, we chose the genes encoding the cytokine interleukin 6 *(Il6)* and its receptor *(Il6r)*, the gene encoding C-X-C motif chemokine 5 *(Cxcl5)*, the *Egr1* gene, encoding early growth response 1, a transcriptional regulator of cell stress and inflammation, the *MafF* gene, encoding small Maf (musculoaponeurotic fibrosarcoma) protein F, a transcriptional regulator of the oxidative stress response that interacts with Nrf2, the *Ho1* (heme oxygenase 1) gene, encoding an anti-inflammatory and anti-oxidant factor, the *Zfp36* (zinc finger protein 36) genes, encoding ARE (AU-rich)-dependent regulators of mRNA stability that primarily target mRNAs involved in the regulation of inflammatory processes, such as transcripts encoding cytokines, and *Atf3* (activating transcription factor 3), encoding a transcriptional repressor of inflammation and promoter of regeneration processes. With regard to mitochondria / metabolism, we focused on *Ppargc1a*, encoding Pgc-1α (peroxisome proliferator-activated receptor gamma coactivator 1-alpha), a transcriptional regulator of oxidative metabolism and mitochondrial biogenesis, *Nr4a3* (nuclear receptor subfamily 4 group A member 3), encoding a transcriptional regulator of inflammation and metabolism, and *Ppp1r3* (protein phosphatase1 regulatory subunit 3A), encoding a regulator of glycogen synthesis. In addition, we analyzed expression of genes encoding different myosin heavy chain isoforms (*MyH* genes), which are characteristic for different muscle fiber types, such as ‘red’, oxidative or ‘white’, glycolytic fibers, and of genes encoding markers of sarcomeric protein decay and processes of sarcomere rearrangement (*MuRF1*, encoding muscle ring finger protein 1, and *Atrogin 1*).

In addition, recent work suggests that small, regulatory RNAs (microRNAs, miRs) play a major role in exercise adaptation. Against this background, we also analyzed expression of a set of selected miRs known to be differentially expressed in skeletal muscle in response to exercise. Specifically, the muscle-specific ‘myomiRs’ miR-133a, -133b, -206, and -208b, as well as -9, -20a, -20b, -21, -23a, -29a, -31 -107, -181a and -378, for which literature data point to an importance in exercise adaptation, were chosen. In addition, we analyzed expression of genes encoding major components of the miR biosynthesis pathways, namely *Xpo5, Dgcr8* (DiGeorge syndrome critical region 8), *Drosha* and *Dicer1*, since differential expression of these genes might indicate activation of miR biogenesis and / or changes in miR expression patterns in general (for review, see [11]).

Our data indicate that PDTC treatment affects a distinct subset, but not all genes regulated by one bout of acute treadmill running in mice.

## Experimental procedures

**Animals.** Male C57BL/6 mice (6–7 weeks old) were purchased from Charles River GmbH (Sulzfeld, Germany). They were housed and fed according to the guidelines of laboratory animal care and all procedures were approved by the local authorities (Regierungspräsidium Tübingen, M9/14). Mice had *ad libitum* access to standard chow (Sniff, Soest, Germany) and tap water and were kept under a 12h-12 h light–dark cycle (light: 5:00 am – 5:00 pm; dark: 5:00 pm – 5:00 am). All treadmill running experiments were performed between 5:00 and 6:00 pm. For the study described here, in total, *n* = 32 mice were used and randomly assigned to one of eight experimental groups (sedentary – sed or exercised – ex, with and without PDTC treatment, either sacrificed immediately after exercise or 3 h later).

**PDTC treatment.** PDTC treatment of mice is an established procedure and has been widely employed prior to this study [8,12–21]. Here, mice were intraperitoneally injected with 20 mg/kg PDTC two hours before training. Control animals were i.p.-injected with the same volume of saline.

**Treadmill running.** Treadmill running was carried out as previously described [22,23]. Briefly, 14 days before the training experiment, mice were allowed to familiarize with the treadmill (Hugo Sachs, March-Hugstetten, Germany) on three individual days for 10 min each. Initially, the treadmill remained switched off, later, speed was gradually increased up to 14 m/min, at 14° inclination. For the actual experiment, mice were 8–9 weeks old. They were initially run at 5 m/min and 5° inclination for 5 min (warm-up), and subsequently for 60 min at 14 m/min and 14° inclination. Mice were sacrificed immediately after the experiment or three hours later, dissected, and individual hindlimb muscles were isolated, snap-frozen in liquid nitrogen, immersed in RNALater®, and stored at −70°C until further use.

**PathScan Akt Array.** To assess activation of signaling cascades in skeletal muscle tissue, the PathScan® Akt Signaling Antibody Array Kit (Cell Signaling Technology, Danvers, MA, USA) was employed according to the manufacturer’s instructions, employing 50 mg of snap-frozen tissue from each animal included in the analysis.

**RNA isolation and qPCR.** RNA isolation was performed using the Fibrous Tissue RNA isolation kit or the miRNA isolation kit (Qiagen). Due to technical issues, a few tissue samples were lost to follow-up or yielded low concentrations of RNA or RNA of insufficient quality, so that for these data points, only results for three mice could be obtained (indicated in the respective figures). Semi-quantitative real time PCR analysis was carried out using the CFX96 touch real time qPCR detection system (Bio-Rad). Gene expression was analyzed using the Eva Green Mastermix (Bio-Rad) or miScript SYBR Green PCR Kit (Qiagen). For detection of different transcripts, pre-designed primers (Qiagen QuantiTect Primer Assays) or self-designed primers (supplementary Tab. 1) were used. In each experiment, melting curve analysis was performed to verify that a single transcript was produced. RT-qPCR relative gene expression was calculated using the comparative CT (2^−ΔΔ*C*^_T_) method, where expression was normalized to *Gapdh, Hprt, Tbp* and *Rps12.* Non-RT- and non-template controls were run for all reactions.

**Statistical analysis.** Statistical analysis was carried out using JMP software (Version 11; SAS Institute, Cary, NC, USA). Data were considered significant with *p*-values of less than 0.5. Data are presented as means +/− SD.

## Results

**PDTC treatment and treadmill running.** The chosen PDTC dosage of 20 mg/kg was well tolerated by all mice. No animal had difficulty in completing the treadmill running session. However, occasionally, mice that sat on the metal bar at the bottom to rest had to be gently tapped on their back to encourage them to resume running.

**Figure 1.**
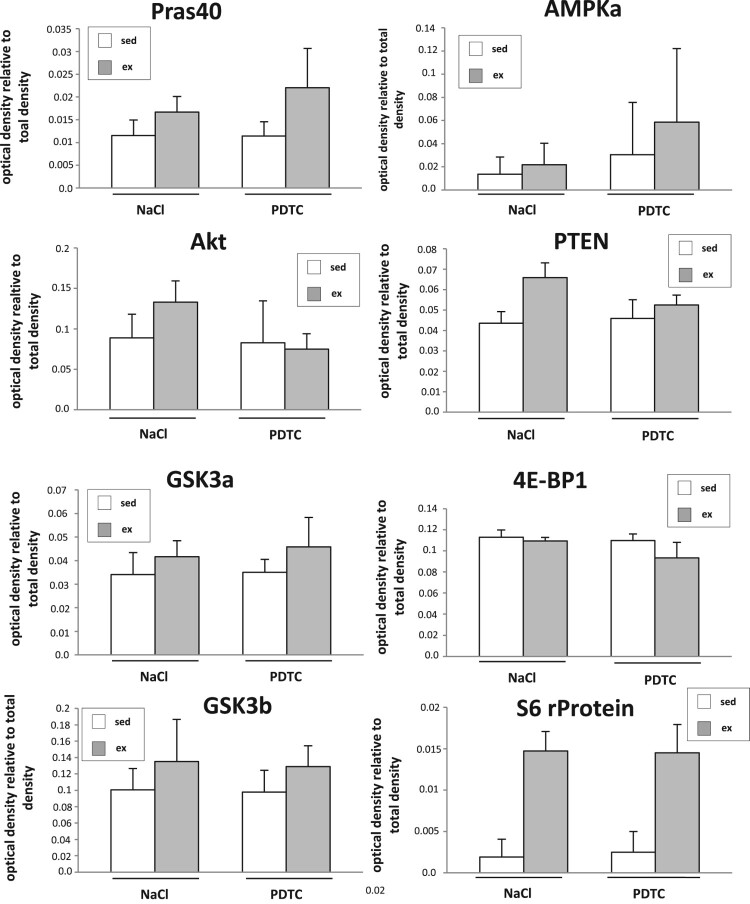
Activation of signal transduction cascades. Densitometric quantification of the results of the PathScan Akt Array (arbitrary units) as indicated. For each experimental group, tissue of *n* = 4 animals was analyzed.

**Activation of signal transduction cascades.** To study activation of signal transduction cascades 3 h after completion of the exercise bout, a commercial pre-designed array (PathScan Akt Array, Cell Signaling Technology) was employed according to the manufacturer’s instructions. As shown in Figure 1, in *M. quadriceps* (Q), besides slight increases in activation of several kinases with running, part of which were sensitive to PDTC treatment, we could particularly detect strongly enhanced activation of S6 ribosomal protein phosphorylation both in the presence as well as in the absence of PDTC. This protein is a downstream target of p70 S6 kinase and reflects mTOR pathway activation, a major regulator of muscle hypertrophy (for review, see [24,25]).

**Figure 2.**
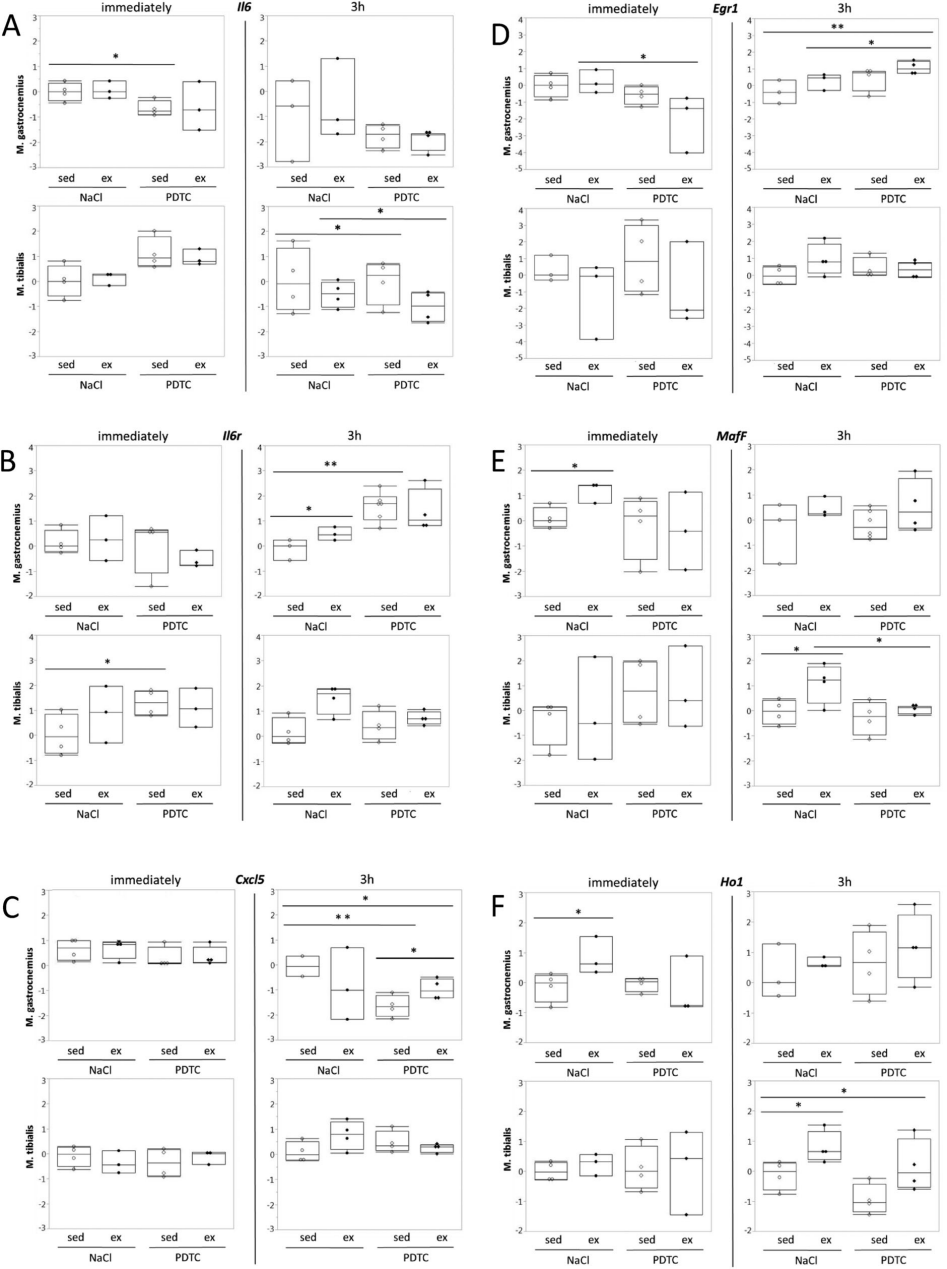
Regulation of genes related to stress and inflammation. Expression of the *Il6* (A), *Il6r* (B), *Cxcl5* (C), *Egr1* (D), *MafF* (E), and *Ho1* (F) genes was analyzed by qPCR as indicated.

**Genes related to cell stress and inflammation.** A major focus of our study was on genes related to cellular stress and inflammation. As shown in Figure 2, interestingly, we could not detect induction of *Il6* expression with exercise. By contrast, at least in *M. tibialis anterior* (TA), we observed a trend towards induction of the gene encoding Il6 receptor (*Il6r*). This effect was more pronounced after 3 h and by then appeared to be repressed by PDTC pre-treatment. Similar trends, at least in TA after 3 h, sometimes also in other muscles and/or immediately after exercise, were observed for other genes encoding stress- and inflammation-associated factors, specifically *Cxcl5, Egr1, MafF*, *Ho1 and Zfp36* (Figure 2 and data not shown). Most of these results, however, did not reach statistical significance.

**Figure 3.**
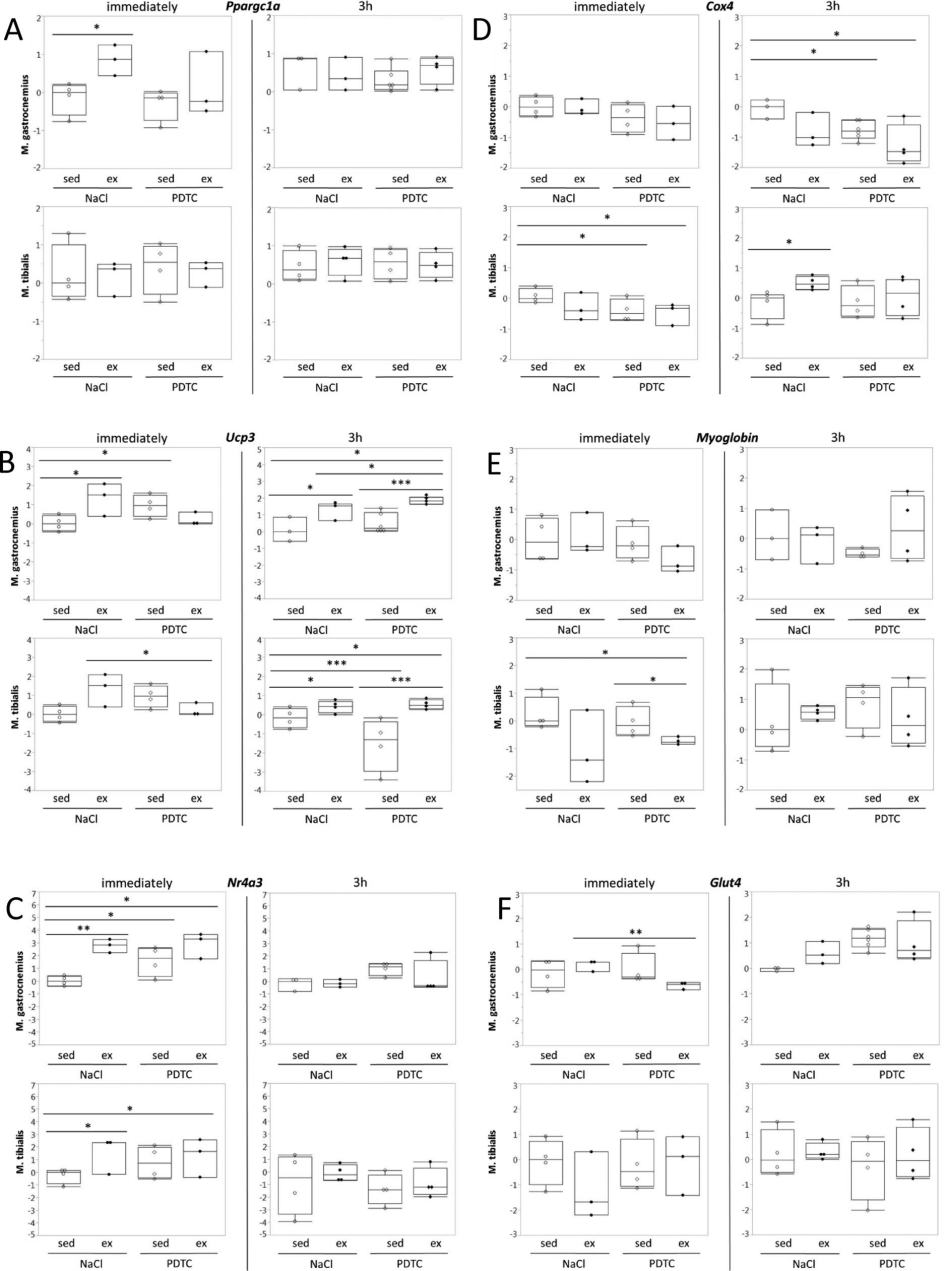
Regulation of genes related to metabolism. Expression of the *Ppargc1a* (A), *Ucp3* (B), *Nr4a3* (C), and *Cox4* (D), *Myoglobin* (E), and *Glut4* (F) genes was analyzed by qPCR as indicated.

**Genes related to metabolism**. When analyzing *Ppargc1a* (encoding Pgc-1α) expression in muscle tissues, we found moderate induction in *M. gastrocnemius* (G), both immediately and after 3 h. Whereas immediate induction was blocked by PDTC pre-treatment, induction after 3 h was not. By contrast, there was little effect in TA. In both muscles, there was a tendency towards higher *Ppargc1a* expression levels after PDTC treatment, at least after 3 h (Figure 3(A)). Quite similar results were obtained for *Ucp3* (Figure 3(B)) and *Nr4a3*, however, in case of the latter, upregulation immediately after exercise was not inhibited by PDTC pre-treatment, and there was also a prominent ‘immediate’ effect in TA (Figure 3(C)). By contrast, there were only minor effects of both exercise and PDTC on expression of *Cox4*, *Myoglobin,* and *Glut4* (Figure 3(D)).

**Figure 4.**
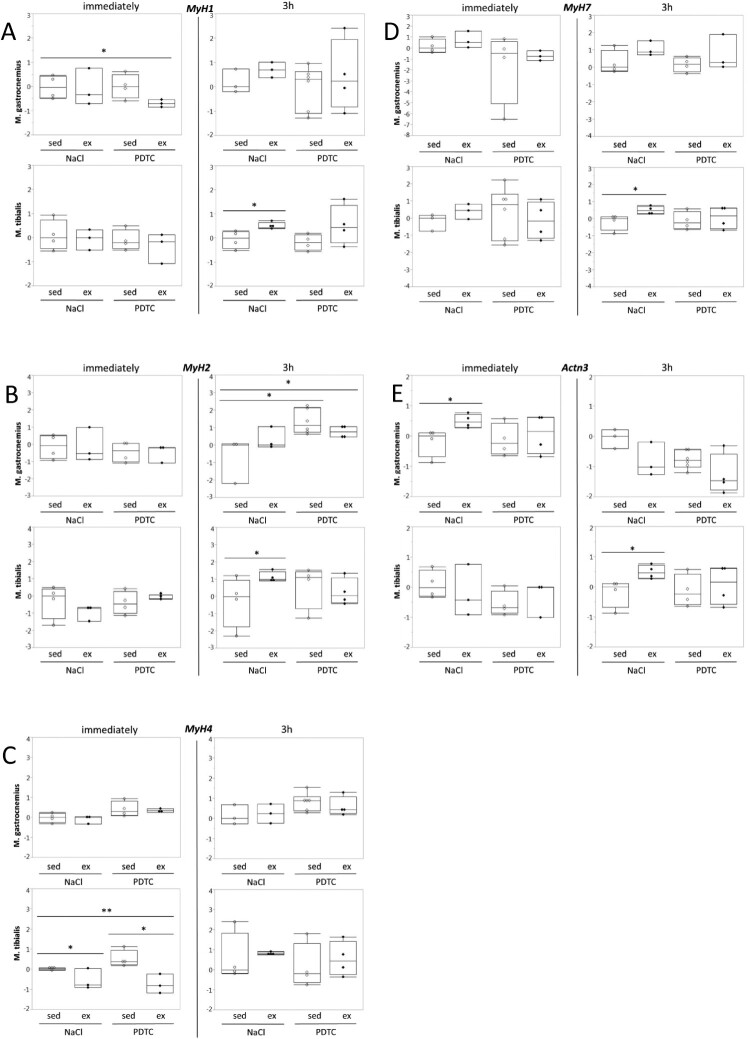
Regulation of genes encoding myosin heavy chain isoforms and actinin 3. Expression of the *MyH1* (A), *MyH2* (B), *MyH4* (C), *MyH7* (D), and *Actn3* (E) genes was analyzed by qPCR as indicated.

**Genes encoding myosin heavy chain isoforms and actinin 3.** Next, we analyzed expression of genes encoding different myosin heavy chain (MHC) isoforms, which are known to be associated with metabolic properties of skeletal muscle fibers. As shown in Figure 4, despite the fact that there were trends towards higher expression of *MyH7*, encoding the ‘oxidative’ MHC1, with running, which appeared to be repressed at least to some extent by PDTC pre-treatment (Figure 4(D)), effects on ‘fast’ and ‘intermediate’ *MyH* genes 1,4 and 2 (encoding MHC2X, 2B and 2A, respectively, Figure 4(A–C)) were inconsistent and diverse, and overall, most of the differences did not reach significance. Furthermore, expression of the *Actn3* gene, which is specific for fast muscle fibers, was hardly altered in response to running and/or PDTC treatment (Figure 4(E)).

**Figure 5.**
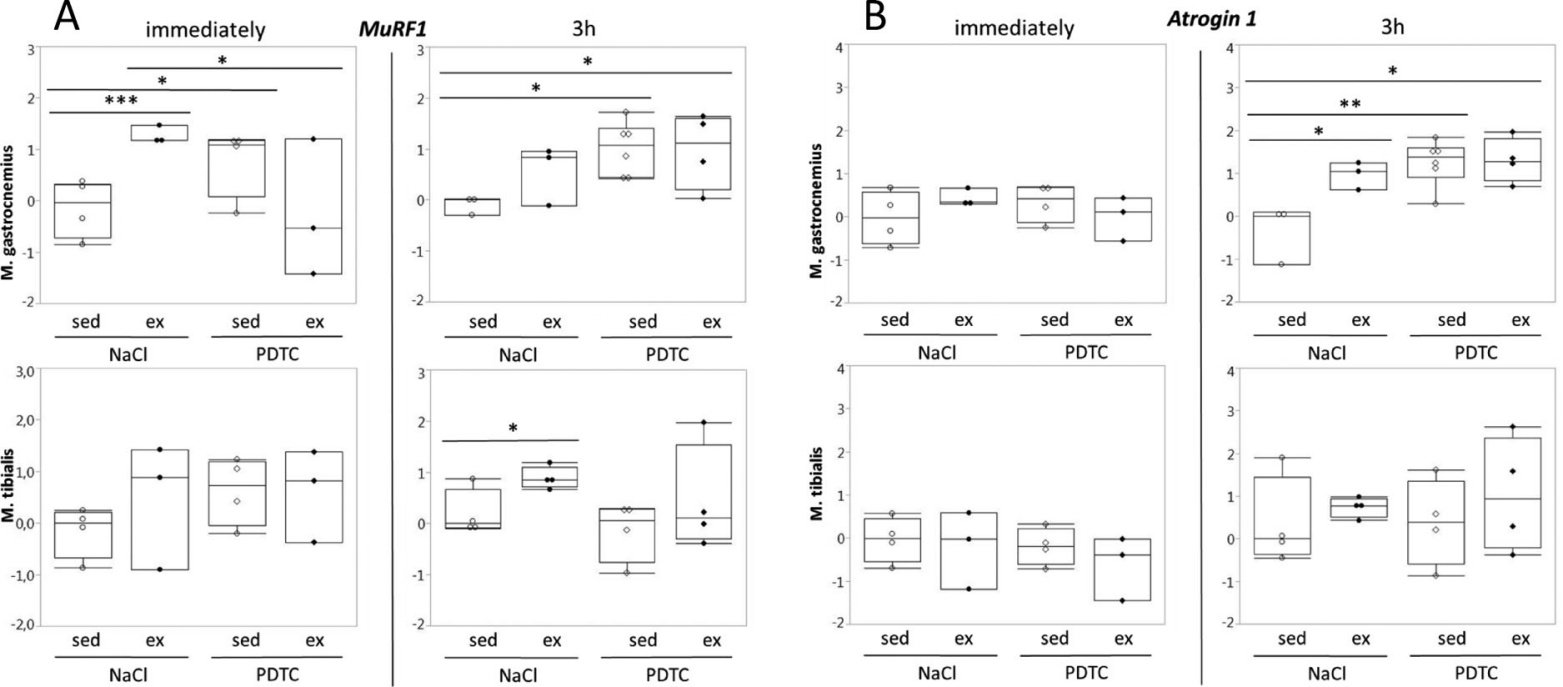
Regulation of genes encoding sarcomere-associated E3 ubiquitin ligases (‘atrogenes’). Expression of the *MuRF1 / Trim63* (A) and the *Atrogin 1 / FBox32* (B) genes was analyzed by qPCR as indicated.

**Genes encoding sarcomere-associated E3 ubiquitin ligases (‘atrogenes’).** Interestingly, expression of *MuRF1 / Trim63*, which encodes a sarcomere-associated E3 ubiquitin ligase involved in the degradation of myosin heavy chain, was induced in response to running in all muscles and at all time points, reaching statistical significance in some cases (Figure 5(A)). The effects of PDTC pre-treatment, by contrast, were variable, however, there was a trend towards *MuRF1* induction by PDTC alone. Similar effects were also observed for the atrogene *Atrogin 1 / FBox32*, specifically in G, however, not until 3 h after exercise (Figure 5(B)).

**Figure 6.**
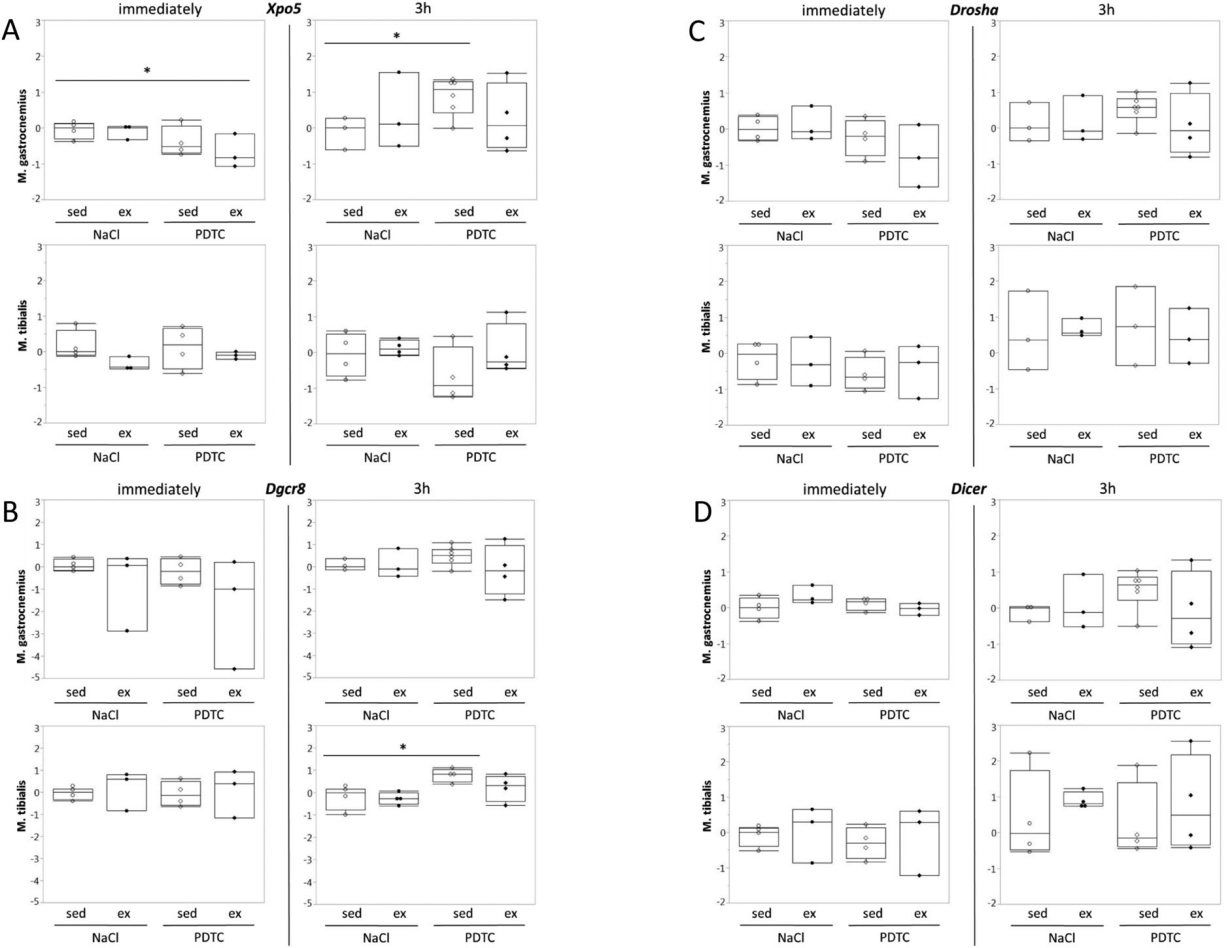
Regulation of genes encoding components of the miR biogenesis pathway. Expression of genes encoding individual components of the miR biogenesis pathway was analyzed by qPCR as indicated.

**Figure 7.**
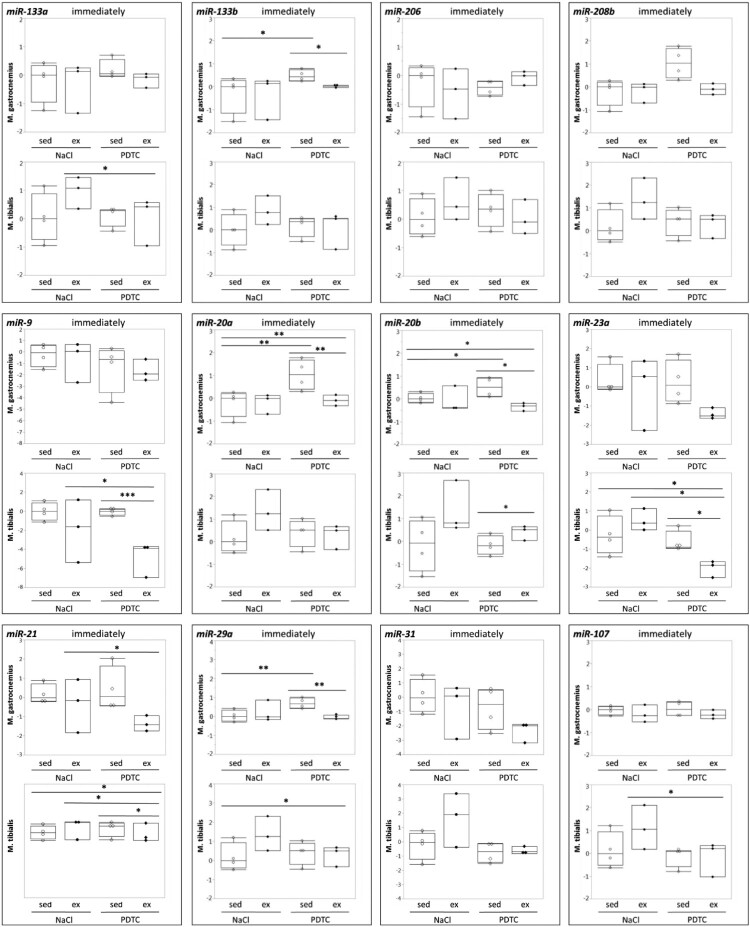
Regulation of selected miRs in response to exercise and PDTC. Concentrations of selected miRs were analyzed by qPCR as indicated.

**Genes encoding components of the miR biogenesis pathway and miRs.** When analyzing regulation of genes encoding components of the miR biogenesis pathway, there were no significant effects (Figure 6(A–D)), suggesting that overall activity of the miR biogenesis pathway was not significantly altered. In addition there were only minor effects on concentrations of individual miRs in response to exercise and/or PDTC (Figure 7 and data not shown), at least immediately after the exercise bout, suggesting that a single bout of moderate running does not immediately change skeletal muscle miR patterns in mice.

## Discussion

Our data demonstrate distinct exercise-induced effects on gene expression, some of which were modulated by PDTC pre-treatment.

Specifically, we found that our running protocol led to phosphorylation of S6 ribosomal protein, most likely by p70 S6 kinase. The latter is usually activated in response to resistance training and/ or amino acid ingestion and signals muscle hypertrophy (for review, see [24,25]). Thus, it is likely that uphill running at moderate intensity, as in our protocol, might not be a mere endurance training, but also induce pathways of skeletal muscle anabolism.

Interestingly, we could not detect induction of *Il6* expression in skeletal muscle tissue. This might be due to specific expression kinetics of this gene, since Merle et al. [26], also failed to observe enhanced concentrations of Il6 mRNA in various murine muscle tissues immediately after a single bout of treadmill running. Furthermore, based on *Il6* expression kinetics in human skeletal muscle ([27], and references therein), it is likely that the time points we chose for our analysis, 0 and 3 h, might have missed the rise, peak and decline of Il6 mRNA. Nevertheless, we observed upregulation of other genes related to cell stress and inflammation, such as *Il6r, Cxcl5, Egr1, MafF,* and *Ho1*.

With regard to metabolism, as expected, we found elevated levels of genes encoding oxidative metabolic regulators, namely Pgc-1α (encoded by the *Ppargc1a* gene), Ucp3, and Nr4a3, at specific time points and in specific muscles, some of which were inhibited by PDTC pretreatment. Similarly, we observed trends towards enhanced expression of the ‘slow’, type I *MyH7* with exercise, which appeared to be inhibited by PDTC, at least in certain muscles and at certain time points, while effects on ‘fast’ *MyH* genes were heterogeneous. Despite the fact that overall, these effects were subtle and sometimes inconsistent, our data suggest that our running protocol induces specific aspects of ‘oxidative’ metabolic adaptation in skeletal muscle.

As described earlier (for review, see [28]), genes encoding the so-called ‘atrogenes’ MuRF1 / Trim63 and atrogin 1 / FBox32 are temporarily induced by exercise, especially by single, unaccustomed bouts, whereas trained individuals tend to show lower expression levels of these genes when compared to sedentary controls. Obviously, unaccustomed bouts of exercise lead to sarcomere ruptures, causing the necessity to degrade damaged myofibrils. Correspondingly, we observed prominent induction of the ‘atrogene’ *MuRF1 / Trim63* with exercise, whereas PDTC appeared to evoke high expression levels of this gene *per se*. This latter result might reflect effects of PDTC on further pathways, for example the calcineurin-FoxO way which is known to induce *MuRF1* expression ([29,30]). Similar trends were observed for another ‘atrogene’, atrogin1 / FBox32, although at later time points.

Surprisingly, we did not find major effects on the genes encoding the main components of the miR synthesis route (for review, see [11]), suggesting that overall activity of this pathway was not significantly altered in response to exercise. Similarly, despite the fact that exercise has been shown to influence concentrations of a broad spectrum of miRs, with regard to the subset analyzed here, we did not find major effects. One explanation for this finding might be the fact that miR regulation, is, in most cases, quite short-lived, suggesting that more time points should have been analyzed. In contrast to our study, most authors analyzed miR patterns several hours after the exercise bout (for review, see [11], and references therein), which might explain this discrepancy. In addition, since most miR analyses on skeletal muscle published so far employed quite vigorous exercise regimens (for review, see [11]), it is well possible that treadmill running as described here was not strenuous enough to bring about major changes in miR concentrations. Thus, in the future, miR transcriptome analysis, preferably on several time points and in connection with more intense exercise regimens, should be carried out.

Obviously, in the two different muscle types, we found consistent, but also diverging effects of both exercise and PDTC. This might reflect their different fiber type composition and thus metabolic situation, but probably also their different loading and strain with uphill running.

So far, we are not able to attribute the observed PDTC effects to a specific signaling pathway. As mentioned above, PDTC exerts multiple direct and indirect effects on several different signaling pathways: As an anti-oxidant, it can act at multiple levels, for example by affecting mitochondrial adaptation in response to endurance exercise [20,31]. This might explain the PDTC effects on exercise-induced expression of ‘metabolic’ and ‘mitochondrial’ genes, such as *Ppargc1a*. Furthermore, anti-oxidants are known to modulate a plethora of known ROS-activated pathways, such as the Nrf2 or MAPK routes, suggesting multiple mechanisms by which PDTC might influence differential gene expression in exercising skeletal muscle.

Of particular interest for a long time has been the fact that PDTC can inhibit activity of the transcription factor NFκB, a major player in skeletal muscle adaptation and remodeling processes, which is known to be activated in response to single bouts of exercise (for review, see [2]). So far, however, little is known on a possible role of NFκB in mediating exercise-induced molecular adaptation of skeletal muscle tissue. Remarkably, however, multiple potential NFκB target genes encode proteins known to be involved in training adaptation, such as pro-inflammatory cytokines, growth factors and their receptors, or metabolic enzymes (for review, see [2]). Thus, it is not unlikely that the PDTC effects we observed might be based on NFκB inhibition. However, we could not detect elevated levels of the NFκB inhibitor protein IκBα, whose concentration shows an inverse correlation with NFκB activity, in our muscle samples, a finding that suggests that the NFκB pathway plays no major role with regard to the PDTC effects we observed. Nevertheless, one should consider that (1) NFκB activity in resting skeletal muscle is probably quite low, making it unlikely that PDTC would be able to exert a major, additional inhibitory effect here, and (2) NFκB activation in response to exercise appears to be rather short-lived, suggesting that potential PDTC effects on IκBα levels would have been observed at an earlier time point, perhaps while the training session was still ongoing [2,32–34].

Overall, our data suggest that PDTC inhibits a distinct, specific subset of exercise-regulated genes in murine skeletal muscle and not the adaptation process to exercise in general. Our study is limited by (a) the small sample size, and also (b) the limited number of marker genes that were analyzed. Future studies should also focus on long-term physiological and functional training aspects, which would allow quantification and evaluation of the adaptation process, not only of skeletal muscle tissue, but of the whole body. In particular, gains in aerobic capacity, i.e. maximum oxygen uptake after several weeks of regular training, should be compared with and without PDTC treatment.

In addition, our experimental system could serve as a model in the future, which might allow testing and comparing the effects of different anti-oxidant and/or anti-inflammatory compounds, and also different types and intensities of training, in a standardized manner.

## Supplementary Material

Supplemental Material
