# Supplementary material for: Effects of the anti-oxidant PDTC in combination with a single bout of treadmill running on murine skeletal muscle
Source: Redox Rep. 2020 Aug 18;25(1):70–9. doi: 10.1080/13510002.2020.1807088 (PMC7480603; doi:10.1080/13510002.2020.1807088)
Supplement: Supplemental Material [file YRER_A_1807088_SM9360.docx]

**gene expression analysis**

| **gene name** | **forward primer (5´->3´)** | **reverse primer (5´->3´)** |
| --- | --- | --- |
| **Cox4** | CGCTCGTTCTGATTTGGGAG | GGCCTTCATGTCCAGCATTC |
| **Cxcl5** | CAAGCAGAACTGAACTACCAT | ATGAACTCCCTGCTTTGATGA |
| **Nr4a3** | AGATACCCTCCAGATATGCCCT | TGGTCAGCTTGGTGTAGTCG |
| **MYH1** | AAGGAGCAGGACACCAGCGCCCA | ATCTCTTTGGTCACTTTCCTGCT |
| **MYH2** | GCTTCAAGTTTGGACCCACG | ACTTCCGGAGGTAAGGAGCA |
| **MYH4** | AGAGCCAAGAGGAAACTGGAGG | TCGTCCTCAATCTTGCTCTGC |
| **MYH7** | GCTGGAAGATGAGTGCTCAGAG | TCCAAACCAGCCATCTCCTCT |
| **IL6** | TCTGCAAGAGACTTCCATCCA | TCCACGATTTCCCAGAGAAC |
| **Egr1** | CCTGACCACAGAGTCCTTTTC | ACCCAACAGTGGCAACACTT |
| **MafF** | GTCTCCAGCCCAGAGGTCATC | CAACTCGCGCTTGACCTTCAG |
| **Atf3** | CTTCCCCAGTGGAGCCAATC | CATTTTGCTCCAGTCTTCGCTC |
| **Ho1** | AGGCTAAGACCGCCTTCCTG | AGCAGGCCTCTGACGAAGTG |
| **Ppargc1α** | GCTCATTGTTGTACTGGTTGGATATG | CGTAGGCCCAGGTACGACAG |
| **Ucp3** | AACCCAGGGGCTCAGAGCGT | GTCCGCTCCCTTGGGGGTGT |
| **Actn3** | CCCTCAGTTCGCAGGACATC | CCAGCTCCTCCTGCAGTGTC |
| **Murf1** | GCAGCTCATCAAGAGCATTGT | CCAAAGTCAATGGCCCTCAA |
| **Fbox32** | GTGAGGACCGGCTACTGTGG | CAATCCAGCTGCCCTTTGTC |
| **Myoglobin** | CCCTGGAGGGTTGAGCACGGT | AGGCCACCTGGTCCTGAAGGG |
| **Xpo5** | CCGTGCACGAATGAGCTTTT | AGGGGTTACGGAAGATGGGA |
| **DGCR8** | GGCGCCACAGGTGGAA | TACACACTGGCGGCTTA |
| **DICER** | CTGAGCTTAGGAGATCCGAGG | CTTCCACGGTGACTCTGACC |
| **DROSHA** | TCTCTGTAGAGACTGTGAATCCTG | GCTACATCTTCCGCTCACGA |

**miRNA analysis**

| **miRNA-ID** | **miRBase accession number** | **primer sequence (5’-3’)** | **bp primer** |
| --- | --- | --- | --- |
| mmu-miR-107-3p | MIMAT0000647 | AGCAGCATTGTACAGGGCTATCA | 23 bp |
| mmu-miR-133a-3p | MIMAT0000145 | TTTGGTCCCCTTCAACCAGCTG | 22 bp |
| mmu-miR-29a-3p | MIMAT0000535 | TAGCACCATCTGAAATCGGTTA | 22 bp |
| mmu-miR-20a-5p | MIMAT0000529 | TAAAGTGCTTATAGTGCAGGTAG | 23 bp |
| mmu-miR-20b-5p | MIMAT0003187 | CAAAGTGCTCATAGTGCAGGTAG | 23 bp |
| mmu-miR-206-3p | MIMAT0000239 | TGGAATGTAAGGAAGTGTGTGG | 22 bp |
| mmu-miR-208b | MIMAT0004939 | ATAAGACGAACAAAAGGTTTGTAAA | 25 bp |

The following primers were purchased from Qiagen (Hilden): ZFP36/Tis11 (Mm_Zfp36_2_SG

= QT01060962), ZFP36 I1/ Tis11b (Mm_Zfp36I1_1, QT00287056), ZFP36I2/Tis11d (Mm_Zfp36l2_2_SG = QT01162000), Mm_miR-133b_1 (MS00007301), Hs_SNORD95 (MS00033726_11), Hs_SNORD96A_11 (MS00033733),Hs_RNU6-2_11(MS00033740), Mm_miR-9_1 ( MS00005887), Mm_miR-181a_2 ( MS00011263), Mm_miR-31_1 ( MS00011760), Mm_miR-23a_2 ( MS00032599), Mm_miR-378_1 ( MS00032788), Mm_miR-21_2 (MS00011487)

Tab.1
